# Comparing the Conventional and Balloon-Guided Catheter-Assisted SWIM Technology for the Treatment of Acute Ischemic Stroke

**DOI:** 10.3389/fneur.2022.866673

**Published:** 2022-07-13

**Authors:** Zhengwen Chen, Yizhi Liu, Bo Li, Chen Yuan, Kaiwen Hou, Long Chen, Peicheng Li

**Affiliations:** Department of Interventional Radiology, The First Affiliated Hospital of Soochow University, Suzhou, China

**Keywords:** ischemic stroke, Mechanical Thrombectomy, balloon-guided catheter, propensity matching, acute ischemic stroke

## Abstract

**Objective:**

Acute ischemic stroke is common in elder patients. This study investigates whether using the balloon-guided catheter (BGC) would improve the effect of stent thrombectomy (Solitaire FR With Intracranial Support Catheter for Mechanical Thrombectomy, SWIM) for patients with acute ischemic stroke due to large vessel occlusion (AIS-LVO).

**Method:**

The data of 209 patients with AIS-LVO underwent SWIM were collected retrospectively from January 2017 to June 2021. These patients were divided into two groups based on whether they used of BGC or not. The propensity score matching (PSM) analysis was used to compare the differences in the first pass effect (FPE), successful recanalization, embolus escape rate, symptomatic intracranial hemorrhage (sICH), 90-day clinical favorable outcome, 90-day all-cause mortality, and complications in the patients treated with SWIM combined with balloon-guided catheter or conventional catheter.

**Results:**

Among the 209 patients, 44 patients were treated with BGC and 165 patients were not. After matching, a total of 111 patients were included. The results showed that there was no statistical difference in FPE (35.1% in non-BGC group compared to 24.3% in BGC group, matched RR, 0.59; 95% CI, 0.24–1.44), successful recanalization (89.2 vs. 91.9%, matched RR, 1.37; 95%CI, 0.34–5.51), embolus escape (6.8 vs. 8.1%, matched RR, 1.22; 95%CI, 0.28–5.40), sICH (8.1 vs. 13.5%; matched RR, 1.77; 95%CI 0.50–6.24), 90-day clinical favorable outcome (48.7 vs. 54.1%, matched RR, 1.11; 95%CI 0.51–2.46), 90-day all-cause mortality (17.6 vs. 21.6%, matched RR, 1.29; 95%CI 0.48–3.47), and the incidence of complications (6.8 vs. 5.4%, matched RR, 0.79 95%CI 0.15–4.27). These results indicate that using SWIM as the first-line treatment for patients with AIS-LVO, there is no statistical significance in FPE, final successful recanalization, distal emboli, sICH, procedural time, 90-day favorable outcome, 90-day mortality, and complications with or without BGC.

**Conclusion:**

Balloon-guided catheter does not affect the result of using SWIM as the first-line treatment for patients with AIS-LVO. Our results will guide daily practice, with the adoption of the use of a guided catheter without a balloon.

## Introduction

Mechanical thrombectomy (MT), represented by stent retriever thrombectomy (SRT), has become the standard of care for acute ischemic stroke due to large vessel occlusion (AIS-LVO) because of the positive results of several randomized control trials (RCTs) in 2015 (1–5). The successful recanalization rate through MT can be up to 90%, but the 90-day clinical favorable outcome is only about 50% (6). Several studies have reported that the first pass effect (FPE), which refers to achieving complete recanalization after one single pass without rescue therapy, is an important factor that affects the prognosis of patients (7, 8). However, embolus escape during MT not only affects the FPE, but also increases the reperfusion time. Therefore, how to avoid thrombus fragmentation has become a new metric for technically successful MT. In this setting, several studies have analyzed the impact of BGC usage on SRT, which showed that BGC can reduce the number of stent passages, decrease the incidence of distal emboli, and shorten the procedure time (9, 10).The guidelines also recommend the use BGC in SRT (class IIa; level of evidence C) (11).

However, the added value of BGC has only been reported in retrospective studies, and most of the first-line modality is SRT (12, 13). With the rapid evolution of endovascular devices, varying thrombectomy techniques, such as Solitaire FR With Intracranial Support Catheter for Mechanical Thrombectomy (SWIM) and continuous aspiration prior to intracranial vascular embolectomy (CAPTIVE), are being used in clinics and have been proved to effectively improve the FPE and the clinical outcomes (14). However, the impact of BGC use in these combined strategies is not clear. Therefore, the primary aim of this study was to compare the reperfusion results and clinical outcomes of AIS-LVO, with and without BGC use, when SWIM is used as the first-line treatment.

## Materials and Methods

The study was conducted in accordance with the Declaration of Helsinki (as revised in 2013) and was approved by the Ethics Committee of the First Affiliated Hospital of Soochow University. All participants provided an informed consent agreement.

We retrospectively analyzed patients with AIS-LVO who were subjected to MT in our institution between January 2017 and June 2021. The inclusion criteria of this study are as follows: (1) age≥18 years; (2) acute ischemic stroke due to LVO of intracranial internal carotid artery (ICA), M1, proximal M2 confirmed by either computed tomographic angiography (CTA), magnetic resonance angiography (MRA), or digital subtraction angiography (DSA); and (3) National Institutes of Health Stroke Scale (NIHSS) score ≧6 points. Exclusion criteria are as follows: (1) the presence of hemorrhage on CT scan; (2) Alberta stroke program early computed tomography scores (ASPECTS) <6; (3) premorbid modified Rankin scale (mRS) score >1; and (4) patients who did not use SWIM as first-line treatment. A total of 209 patients were collected, including 44 patients in the BGC group and 165 patients in the non-BGC group.

### Endovascular Procedure

All intervention procedures were performed by two neuro-interventionists with more than 5 years of experience. According to the guideline (11), patients who meet the criteria will receive intravenous (IV) alteplase [recombinant tissue plasminogen activator (r-tPA)] at a dose of 0.9 mg/kg on admission. All treatments were performed either under conscious sedation or general anesthesia. It is up to the operator's discretion to decide whether to use BGC or not. Using Siemens Artis Zeego or Toshiba INFX-8000V DSA as guidance equipment and 8F catheter sheath (Terumo, Japan) was placed through the femoral artery, and the whole brain angiography was performed to identify the site of occlusion.

An 8F BGC (FG2 or Merci) or 8F conventional catheter (Neuromax, Penumbra, USA) was positioned at the extracranial segment of the occluded ICA, then, an intracranial support catheter (Navien, EV3, USA) was introduced, and a microcatheter (Rebar 18 or 27, EV3, USA) was placed over the microwire (Transend-PLATINUM,Boston Scientific, USA) of the occluded area. Under the road map, the stent (Solitaire AB, ev3, Irvine, California, USA) was advanced to the occluded segment and withdrew the microcatheter to deploy the stent for 5 min. Subsequently, the stent was partially retrieved by microcatheter and pushed the intracranial support catheter (Navien, EV3, USA) to the proximal end of the stent. The stent, microcatheter, and intracranial support catheter were gently pulled back to the guide catheter as a unit. In the BGC group, before stent retrieval, the balloon of the BGC was inflated to arrest the antegrade ICA flow. After the stent was retrieved, the balloon was immediately deflated to allow ICA re-circulation.

Follow-up angiography was conducted immediately. If blood flow returned to modified thrombolysis in cerebral infarction (mTICI) ≥2b and could still maintain mTICI ≥2b after 15 min, the procedure was ended. However, if retrieval failed or recanalization was insufficient (mTICI <2b), the procedure would be repeated, but the total number of thrombectomy was not more than 4 times. If the target segment stenosis was >70% or cannot maintain blood flow, rescue therapy, such as balloon angioplasty or stent implantation, would be carried out.

All patients underwent brain CT immediately and 24 h after MT to evaluate hemorrhagic complications. Additional imaging was performed at any time if neurological deterioration appeared. We used the ECASSIII criteria of symptomatic intracranial hemorrhage (sICH) defined as any new intracranial hemorrhage on follow-up imaging with an increase in the NIHSS score by 4 within 24 h.

### Outcome Measurement

Clinical outcome was assessed using the modified Rankin scale (mRS) at 90 days by clinical visit, which was assessed by a neurologist or certified research nurse in our core laboratory. The primary outcome of this study was FPE defined as mTICI 2c-3 of reperfusion after a single attempt of thrombectomy. The secondary outcomes included successful recanalization rate defined as mTICI 2b-3 of reperfusion on the final angiography; embolus escape (defined as angiographic distal branch occlusion of the same or a new vascular territory) (15); the clinical favorable outcome (defined as mRS score of ≦2 at 90 days); sICH; 90-day all-cause mortality; and procedural complications (defined as target vessel perforation, dissection, or spasm).

### Statistical Analysis

All statistical analyses were conducted using the SPSS software (version 23.0) and R statistical software (version 3.6.0). Baseline data of categorical variables were described using frequencies and percentages, whereas continuous variables were described using mean ± SD or median [interquartile range (IQR): 25–75%) as appropriate. For adjusting between-group differences, the propensity scores (PS) were developed to reflect the probability of each patient receiving BGC. Patients included in the BGC group were matched 1:2 to patients in the non-BGC group according to PS using the greedy nearest neighbor matching algorithm with a caliper width of 0.2 standard deviations of logit for PS. The specific matching dimensions are listed in Table 1. The outcomes on this well-balanced group were compared. The Pearson chi-square test or Fisher's exact test was used for categorical variables, and the independent-samples *t*-test or Mann–Whitney *U*-test was used for continuous variables. A two-sided *p*-value of <0.05 was considered statistically significant in all analyses.

**Table 1:** Demographic, clinical, procedural characteristics, and outcomes according to first-line treatment strategy (BGC vs. non-BGC) in the overall population and the matched cohort.

	**Unadjusted**		***p*-value**	**Matched**		***p*-value**
	**BGC** **(*n* = 44)**	**SWIM** **(*n* = 165)**		**BGC** **(*n* = 37)**	**SWIM** **(*n* = 74)**	
**Demographics and medical history**
Age, yr mean±SD	65.4 ± 1.5	67.9 ± 0.6	0.12	67.7 ± 1.2	65.9 ± 0.9	0.25
Male	23 (52.3)	97 (58.8)	0.53	20 (54.1)	43 (58.1)	0.68
Hypertension	29 (65.9)	107 (64.8)	0.92	26 (70.3)	48 (64.9)	0.57
Diabetes	7 (15.9)	53 (32.1)	**0.03**	7 (18.9)	10 (13.5)	0.46
Hypercholesterolemia	6 (13.6)	22 (13.3)	0.99	4 (10.8)	8 (10.8)	>0.99
Atrial fibrillation	17 (38.6)	58 (35.2)	0.77	16 (43.2)	23 (31.1)	0.21
Current smoking	7 (15.9)	52 (31.5)	**0.04**	7 (18.9)	15 (20.3)	0.87
History of stroke	4 (9.1)	17 (10.3)	0.77	4 (10.8)	10 (13.5)	0.69
**Etiology**
Cardioembolic	18 (39.1)	47 (30.9)	0.55	13 (35.1)	23 (31.1)	0.73
ICAD	22 (47.8)	79 (52.0)		20 (54.1)	39 (52.7)	
Others	6 (13.1)	26 (17.1)		4 (10.8)	12 (16.2)	
**Clinical and procedural characteristics**
NIHSS score, median (IQR)	16 (11–19)	14 (10–18)	0.31	16 (11–19)	13.5 (10–18)	0.30
ASPECTS, median (IQR)	9 (8–10)	9 (8–10)	0.70	9 (7–10)	9 (8–10)	0.57
**Site of occlusion**
ICA	14 (31.8)	68 (41.2)	0.66	13 (35.1)	21 (28.4)	0.55
MCA-M1	28 (63.6)	91 (55.2)		22 (59.5)	51 (68.9)	
MCA-M2	2 (4.5)	6 (3.6)		2 (5.4)	2 (2.7)	
Admission SBP, mmHg, mean ± SD	134.1 ± 2.7	135.7 ± 1.8	0.19	133.9 ± 2.9	139.4 ± 3.7	0.09
IV-tPA	32 (72.7)	91 (55.2)	**0.03**	27 (73.0)	52 (70.3)	0.77
General anesthesia	28 (63.6)	75 (45.5)	**0.05**	21 (56.8)	44 (59.1)	0.43
Onset to image time, min (IQR)	200 (134–298)	210 (139–300)	0.46	180 (126–255)	193 (132–293)	0.87
Onset to groin puncture, min (IQR)	305 (263–421)	340 (240–445)	0.24	300 (250–405)	346 (244–434)	0.17
Number of passes (IQR)	2 (1–3)	2 (1–3)	0.98	2 (1–3)	2 (1–4)	0.29
Rescue strategy	6 (13.0)	37 (24.3)	0.10	6 (14.0)	9 (20.9)	0.79
**Outcome**
FPE (mTICI2c-3)	13 (29.5)	51 (30.9)	0.78	9 (24.3)	26 (35.1)	0.25
Successful recanalization (mTICI2b-3)	40 (90.9)	151 (91.5)	0.94	34 (91.9)	66 (89.2)	0.65
Distal emboli	4 (9.1)	17 (10.3)	0.77	3 (8.1)	5 (6.8)	0.80
sICH	6 (13.6)	19 (11.5)	0.75	5 (13.5)	6 (8.1)	0.37
Procedural time	150 (112–180)	140 (100–180)	0.40	150 (115–180)	120 (90–180)	0.13
90-day mRS 0–2	26 (59.1)	84 (50.9)	0.43	20 (54.1)	38 (48.7)	0.79
90-day mortality	9 (19.6)	23 (15.1)	0.47	8 (21.6)	13 (17.6)	0.61
Complication	3 (6.5)	10 (6.6)	0.99	2 (5.4)	5 (6.8)	0.78

*ICAD, Intracranial atherosclerotic disease; NIHSS, National Institute of Health Stroke Scale; MCA, Middle cerebral artery; ASPECTS, Alberta stroke program early CT score; FPE, First pass effect; sICH, Symptomatic intracranial hemorrhage; mRS, modified Rankin scale. Bold values indicates statistical significance*.

## Results

**Figure 1:**
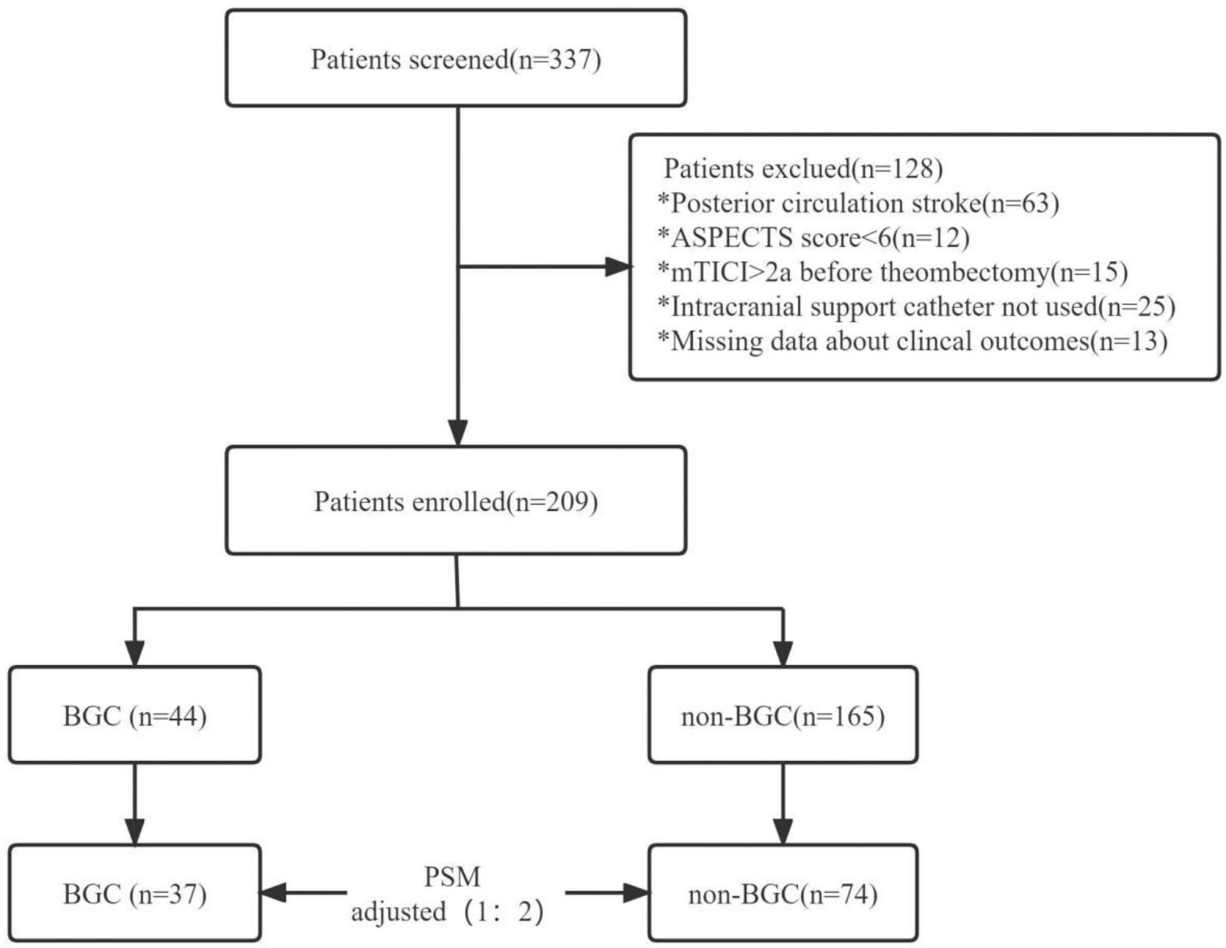
Study flow chart. ASPECTS, Alberta stroke program early CT score; mTICI, modified thrombolysis in cerebral infarction; BGC, balloon-guided catheter; PSM, propensity score matching. *Playing the role of classification and identification.

A total of 337 patients underwent MT were enrolled in the study, and 209 patients were included in the evaluation (Figure 1), with 44 patients in the BGC group and 165 patients in the non-BGC group. After adjustment with the use of PSM, 37 matched pairs were found. Baseline demographics are presented and compared by univariate analysis in both cohorts (Table 1). Before matching, several meaningful differences were found. The patients in the non-BGC group had more diabetes (32.1 vs. 15.9%; *p* = 0.03), current smoking (31.5 vs. 15.9%; *p* = 0.04), but were less commonly administered intravenous r-tPA (IV r-tPA; 55.2 vs. 72.7%; *p* = 0.03) and less likely to choose MT under general anesthesia (45.5 vs. 63.6%; *p* = 0.05) compared with patients in the BGC group. These differences were reduced after PSM.

**Figure 2:**
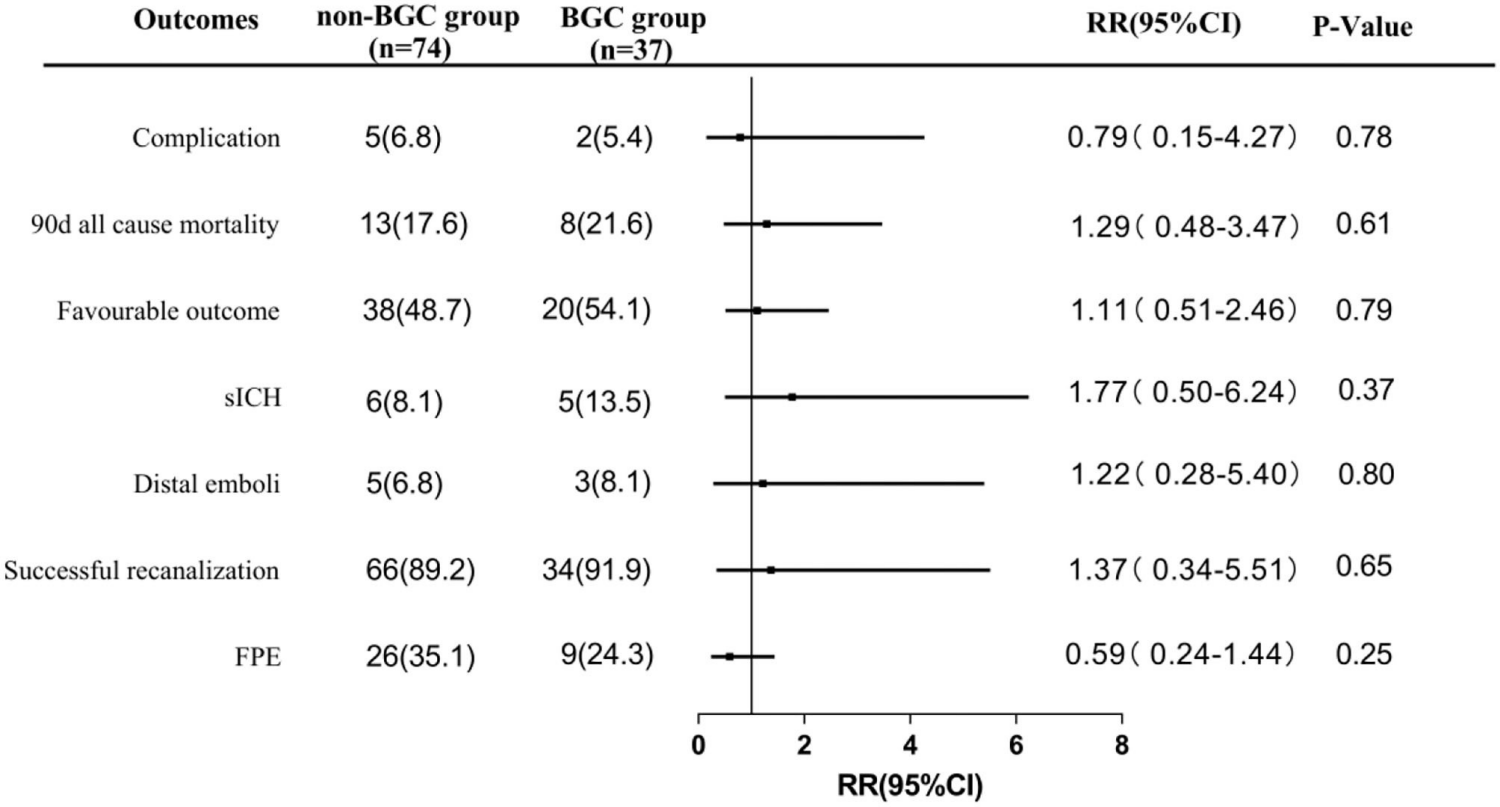
Comparisons in angiographic and clinical outcomes in propensity-score-matched cohort. FPE, first pass effect; sICH, symptomatic intracranial hemorrhage; favorable outcome was defined as 90-day mRs 0 to 2. Complication was defined as vessel perforation, dissection or spasm; RR, relative ratio.

In the propensity score matched cohort, there was no difference in FPE rate between patients treated by first-line SWIM strategy with and without BGC use. About 35.1% of patients achieved FPE in the non-BGC group compared to 24.3% in the BGC group (matched RR, 0.59; 95%CI, 0.24–1.44). The rate of successful recanalization did not differ between the two groups (89.2% in non-BGC vs. 91.9% in BGC group, matched RR, 1.37; 95%CI, 0.34–5.51). Similarly, no significant difference was found in embolus escape rate (6.8 vs. 8.1%,matched RR, 1.22; 95%CI, 0.28–5.40), sICH (8.1 vs. 13.5%, matched RR,1.77; 95%CI, 0.50–6.24), 90-day all-cause mortality (17.6 vs. 21.6%, matched RR, 1.29; 95%CI 0.48–3.47), the incidence of complications (6.8 vs. 5.4%, matched RR, 0.79; 95%CI 0.15–4.27), and 90-day favorable outcome (48.7 vs. 54.1%, matched RR,1.11; 95%CI 0.51–2.46) (Figure 2). These results indicate that using SWIM as the first-line treatment for patients with AIS-LVO, there is no statistical significance in FPE, final successful recanalization, distal emboli, sICH, procedural time, 90-day favorable outcome, 90-day mortality, and complications with or without BGC.

## Discussion

The main finding of this study was that, in patients who underwent intracranial support catheter combined with stent thrombectomy, BGC did not improve the rate of FPE. This was in line with the report by Bourcier et al. (16). They found that balloon-guided catheter is not superior to conventional catheter when stent retriever and contact aspiration are combined for stroke treatment.

Since 2016, Velasco et al. (17) found that the use of the BGC with SRT can increase the FPE, reduce the procedure time, and improve the clinical outcome. BGC is a frequently considered option to modify MT. Kim et al. (18) reported that combination usage of contact aspiration (CA) with BGC can achieve better angiographic performances. ASTER 1 (19) was designed to compare the efficacy CA vs. the SRT and BGC technique as a first-line EVT strategy, which found that there is no difference between these two strategies in successful revascularization rate at the end of the procedure. But the beneficial effect of BGC on combined strategy has not been fully studied. According to the results of our study, combined use of SWIM and BGC did not further improve angiographic outcomes. The possible reason is that SWIM strategy has dual mechanism of “combination of drawing and pulling.” Based on stent thrombectomy, combined with intracranial support catheter suction to achieve the dual protection of “stent grab” and “support catheter suction” (19). Therefore, additional use of BGC will not significantly improve clinical outcomes.

Previous studies have shown that multi-passes and embolus escape seriously affect the MT efficiency (8, 20). As a consequence, different adjunctive techniques, such as BGC, have been implemented to further decrease clot fragmentation and number of passes. During the process of MT, after balloon filling, BGC can temporarily block the forward blood flow and reduce the escape of emboli, stabilize the thrombectomy system, and reduce the number of passes. Theoretically, these two techniques may have synergistic effect. However, the results of this study show that combining these two techniques do not significantly decrease the occurrence of embolus escape (8.1 vs. 6.8%) and number of passes, which is different from the result of using BGC in SRT. We speculated that there are several reasons. First, when the stent is deployed, the thrombus ruptures and flows to the distal region. Because the balloon did not fill when the stent was deployed, distal embolism could occur. Second, a long length of susceptible vessel will collapse caused by BGC aspiration, but the anterior choroidal, ophthalmic, and posterior communicating arteries, which may reverse flow, reduced the suction effect and lead to unintended distal embolization (21). Third, negative suction produces the pressure difference in hydrostatic pressure inside and outside the intracranial support catheter. If BGC is used with SWIM technology, the antegrade blood flow is completely blocked by the filled balloon, and it is no longer easy to produce fluid pressure gradient inside and outside the intracranial support catheter and then partially affect the efficiency of thrombectomy. Of course, these speculative factors need to be verified by more hydrodynamic experiments both *in vitro* and *in vivo*.

Balloon-guided catheter has its disadvantages. First, when using a BGC, we must choose an 8Fr or 9Fr groin sheath, which is larger than the typical 6Fr access. Second, it is difficult to deploy BGC in tortuous arches. Third, BGC is much more expensive than conventional catheter (22). Indeed, BGC has not gained wider clinical use (certainly <50% of MT are performed with a BGC) even in the most recent clinical trials (23). We were unable to record the cases of patients who transfer the first-line strategy due to BGC placement failures. However, we can find that patients treated with BGC were younger than those without BGC (about 2 years younger), suggesting that there was a bias in treating patients with more tortuous arches without the use of a BGC. We thought that it was a limitation of relevant retrospective study. To minimize these confounding factors,we performed PSM and the results were not deviated.

This study has several limitations. First, we cannot avoid all inherent limitations of the retrospective design, although we use PSM analyses to reduce selection bias between the BGC and non-BGC groups, potential flaws may exist in this nonrandomized study. Second, the use of a BGC is not randomized but is used at the discretion of the treating operators, and access difficulty or tortuosity may have precluded the use of BGC in several cases, which may affect the outcomes. Third, we do not exclude the tandem occlusions. It is well known that tandem occlusions are more complex and less likely to achieve FPE. Finally, we do not evaluate the thrombus histology and clot burden, which might associate with FPE and clinical outcomes based on previous studies (24, 25).

## Conclusion

Using SWIM as a first-line strategy for managing AIS-LVO, there is no statistical significance in FPE, final successful recanalization, distal emboli, sICH, procedural time, 90-day favorable outcome, 90-day mortality, and complications with or without BGC. Our results will guide daily practice, with adoption of the use of a guided catheter without a balloon.

## Data Availability Statement

The original contributions presented in the study are included in the article/supplementary material, further inquiries can be directed to the corresponding authors.

## Ethics Statement

The studies involving human participants were reviewed and approved by The First Affiliated Hospital of Soochow University. The patients/participants provided their written informed consent to participate in this study.

## Author Contributions

PL, LC, and ZC contributed to conception and design of the study. YL and BL organized the database. CY and KH performed the statistical analysis. ZC wrote the first draft of the manuscript. ZC and PL wrote sections of the manuscript. All authors contributed to manuscript revision, read, and approved the submitted version.

## Conflict of Interest

The authors declare that the research was conducted in the absence of any commercial or financial relationships that could be construed as a potential conflict of interest.

## Publisher's Note

All claims expressed in this article are solely those of the authors and do not necessarily represent those of their affiliated organizations, or those of the publisher, the editors and the reviewers. Any product that may be evaluated in this article, or claim that may be made by its manufacturer, is not guaranteed or endorsed by the publisher.
